# P-1619. Diagnostic Stewardship: Over-testing for *Herpes Simplex Virus* in Bronchoalveolar Lavage Specimens

**DOI:** 10.1093/ofid/ofae631.1786

**Published:** 2025-01-29

**Authors:** Oliver Lin, Bailey Burnette, Becky Reece

**Affiliations:** West Virginia University, Morgantown, West Virginia; WVU Medicine, Morgantown, West Virginia; West Virginia University, Morgantown, West Virginia

## Abstract

**Background:**

HSV-1 is estimated to affect 3.7 billion worldwide (67% prevalence), primarily manifesting as oropharyngeal lesions. Although HSV can be commonly detected by polymerase chain reaction (PCR) in bronchoalveolar lavage (BAL) specimens, true HSV pneumonitis is rare and most positive specimens reflect colonization or asymptomatic reactivation, not active respiratory infection. This study evaluates the appropriateness and frequency that diagnostic HSV-1/2 qualitative DNA PCRs are ordered on BAL specimens at a tertiary medical center and its impact on treatment and patient outcomes.

Immune Status of Patients Tested for Opportunistic Infections

**Figure:**
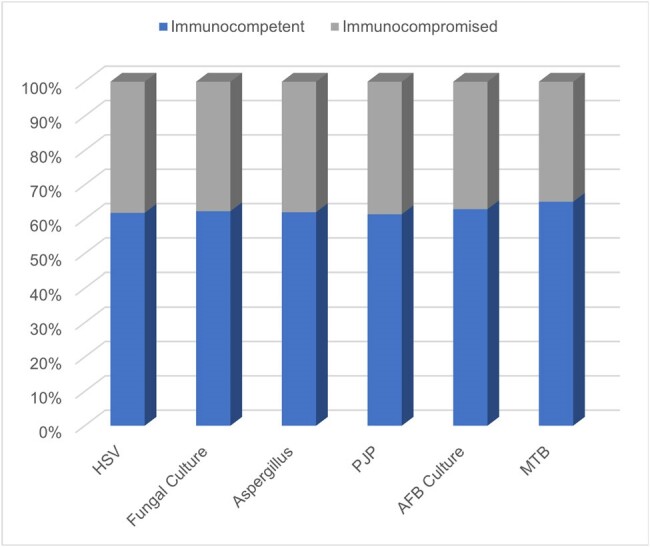

**Methods:**

Patients at a single tertiary medical center who had HSV-1/2 qualitative DNA PCR performed on any specimen in 2022 were identified by lab order. Testing performed on non-BAL specimens were excluded from data collection. De-identified clinical history, risk factors, immune status, concomitant testing, treatment, and outcomes were recorded to secure database for analysis.

Immune Status Guiding Treatment in HSV Positive Patients

**Figure:**
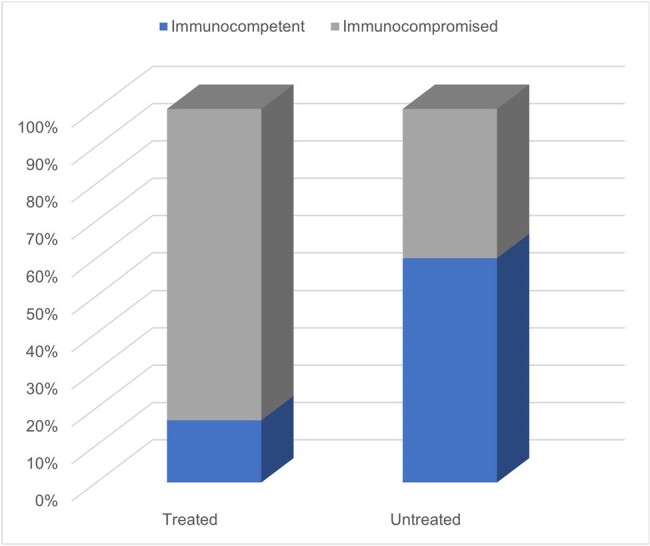

**Results:**

HSV-1/2 qualitative DNA PCR testing was performed on BAL specimens from 131 unique patients. Of the 131 patients, 50 (38.2%) were immunosuppressed as defined by active malignancy, pharmacologic immunosuppression, neutropenia, HIV positive thus most testing was performed on immunocompetent patients without risk factors. 16 patients had a positive qualitative PCR (12.2%) of which 6 patients were treated with antiviral therapy (4.6%). 12 patients (9.2%) were tested more than once on different occasions, 4 of which were positive. 1 patient had a documented final diagnosis of HSV pneumonitis treated with acyclovir.

**Conclusion:**

HSV pneumonitis is a rare manifestation of a common infection that is associated with critical illness, poor outcomes. Many patients will have positive PCR testing of BAL specimens due to inoculation from the upper respiratory tract but this typically does not reflect true infection. Only in the context of significant immunosuppression, compatible clinical presentation can HSV pneumonitis be diagnosed. Excessive ordering of HSV testing leads to unnecessary healthcare expenditure and confounds the clinical picture and decision making. We suggest limiting HSV testing to immunosuppressed patients with the appropriate clinical presentation and use of cytopathology to help confirm diagnosis.

**Disclosures:**

**All Authors**: No reported disclosures

